# Labor induction in pregnancy complicated by myelodysplastic syndrome: A case report

**DOI:** 10.1002/ccr3.3935

**Published:** 2021-02-14

**Authors:** Changsheng Peng, Tao Cui, Qiang Yao

**Affiliations:** ^1^ Department of Gynecology and Obstetrics West China Second University Hospital Sichuan University Chengdu China; ^2^ Key Laboratory of Birth Defects and Related Diseases of Women and Children (Sichuan University) Ministry of Education Chengdu China

**Keywords:** cross‐matched platelets, labor induction, myelodysplastic syndrome, pregnancy

## Abstract

Pregnancy may aggravate myelodysplastic syndrome. Cross‐matched platelets can be used in cases of refractory thrombocytopenia. Vaginal delivery can be attempted if the platelet count is at least 20 × 10^9^/L.

## Graphical abstract

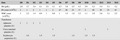

## 1 INTRODUCTION

Myelodysplastic syndrome (MDS) is an extremely rare pregnancy complication that can be particularly complex to manage. Transfusion of cross‐matched platelets may be an effective treatment for patients with antiplatelet antibodies. These patients may benefit from a trial of vaginal delivery if their platelet count is at least 20 × 10^9^/L.

Myelodysplastic syndrome is an extremely rare pregnancy complication that may lead to conditions including maternal hemorrhage, infection, and fetal growth restriction. In women who also suffer from severe anemia and thrombocytopenia, management of myelodysplastic syndrome can be particularly complex. There is currently no standard treatment guideline. This article introduces a case of induction of labor in pregnancy complicated by myelodysplastic syndrome, which presented in the form of refractory thrombocytopenia. The patient clinically presented with multiple spontaneous bleeding throughout the body and severe infection, and the platelet count was maintained at 1‐2 × 10^9^/L after repeat platelet input. Severe thrombocytopenia and infection prevented the continuation of this pregnancy. After discussion with the patient and her family, we decided to terminate the pregnancy, but the process was very challenging mainly due to refractory thrombocytopenia. After multidisciplinary discussion, we chose to perform an induction via vaginal route as we determined that it may be less harmful than a cesarean section. Cross‐matched platelets were transfused to rapidly improve platelet count and perform induction of labor.

## 2 CASE REPORT

A 23‐year‐old patient was admitted to the hospital for "26 + 5 weeks of amenorrhea and 5+ hours of epistaxis." Throughout the pregnancy, the patient repeatedly experienced bleeding from the nose and gingiva, which she managed on her own. She has a personal history of being diagnosed with myelodysplastic syndrome over 10 years ago following a bone marrow biopsy and repeatedly had blood and platelet transfusions. With the long‐term use of oral medications (self‐purchased hormones and traditional Chinese medicine), her condition had been stable. Physical examination revealed temperature of 39.7°C, pulse 127 times/min, respiratory rate 25 times/min, and blood pressure 131/64 mm Hg. The whole body was covered with scattered petechia and ecchymosis, and she also had bleeding gums and intranasal blood clots. Gynecological examination revealed that she had no uterine contraction, but we noted a small amount of bloody secretion in the vagina. Fetal Color Doppler Ultrasound showed an intrauterine single live birth (equivalent to 24 weeks of pregnancy) in breech position, with a single peak in the S/D (Umbilical Systolic pressure/Diastolic pressure of the fetus) value. Laboratory investigations showed a WBC count of 3.0 × 10^9^/L, neutrophils 53.2%, hemoglobin 2.8 g/dL, and platelets 2 × 10^9^/L.

After admission, the patient developed subconjunctival hemorrhage, impaired vision, and increased petechia and ecchymosis. The blood culture indicated Klebsiella pneumonia infection. We administered Jisaixin Recombinant Human Granulocyte Colony‐stimulating Factor Injection, transfused her with 6 U of erythrocyte suspension without WBC and 4 U of apheresis platelet a total of eight times, and administered piperacillin and meropenem against infection. At this point, a routine blood test showed: WBC count 0.9 × 10^9^/L, Hb 4.1 g/dL, Plt count 1 × 10^9^/L. These values indicated that the blood transfusions were not effective. The patient had extremely low platelets, which led us to consider the possibility that these results may be due to the presence of platelet and red blood cell antibodies in her blood vessels. After multidisciplinary discussion involving Departments of Obstetrics, Anaesthesiology, Haematology, Infectious Diseases, and Critical Care Medicine, we transfused her again with 1 U of cross‐matched platelets, 1.5 U of erythrocyte suspension without WBC. Following this round of transfusion, her blood test showed Hb of 4.3 g/dL and a Plt count of 47 × 10^9^/L. Rivanol was then injected into the amniotic cavity, and 72 hours later, the labor process began following the placement of Misoprostol in the vagina for cervix maturity. The patient was re‐transfused with 1 U of cross‐matched platelets and 1.5 U of erythrocyte suspension without WBC half an hour before delivery. To shorten the labor process, a stillbirth was facilitated using breech extraction. The fetus was 36 cm in length, weighed 1010 g, with no obvious abnormality in appearance. The patient had 300 mL of bleeding. Postpartum review of blood routine test showed: WBC count 2.5 × 10^9^/L, Hb 52 g/L, Plt count 43 × 10^9^/L (Table 1). The patient was in a relatively stable condition according to a telephone follow‐up conducted 42 days later.

**TABLE 1:** Blood condition of the patient after admission

Date	D0	D1	D2	D3	D4	D5	D6	D7	D8	D9	D10	D11	D12	D13	D14
Hb (g/dL)	2.8	3.7	3.4	4.1	3.5	4.1	4.3	3.8	4.5	4.3	5.9	4.3	5.4	5.1	5.2
Plt count (×10^9^/L)	2	1	2	1	1	2	47	28	23	17	2	40	38	23	43
WBC count (×10^9^/L)	3	2.2	1.4	0.9	1	0.9	1	1.5	1.8	2.2	1.1	2.9	2.6	3.1	2.5
Transfusion															
Apheresis platelets (U)	1	1	1	1											
Cross‐matched platelets (U)						1					1			1	
Erythrocyte suspension (U)		4.5		1.5		1.5			1.5		1.5	1.5			

Note

We transfused the apheresis platelet 1 U per day for the patient in the first 4 d after admission; then, we gave the cross‐matched platelets on the fifth, tenth, and thirteenth day; we also transfused 4.5 U of erythrocyte suspension without WBC on the first day of admission; then, on the third, fifth, eighth, tenth, eleventh day, we transfused 1.5 U of erythrocyte suspension without WBC each day.

## 3 DISCUSSION

Myelodysplastic syndrome (MDS) is a group of clonal stem cell disorders originating from myeloid stem cells or pluripotent stem cells. Its overall incidence is reported in the literature as 4/100 000, and the incidence gradually increases with age, with only rare cases seen in patients under the age of 40.^1^, ^2^ Main features of MDS include ineffective hematopoiesis, cytogenetic and molecular biological abnormalities, and high‐risk transformation into acute myeloid leukemia (AML), which has variable clinical manifestations and is difficult to diagnose and treat.^3^ Pregnancy complicated with myelodysplastic syndrome is extremely rare, mostly reported in individual case reports,^4^, ^5^, ^6^ and is exceedingly difficult to treat. There is no standardized guideline in its diagnosis and treatment at the national or international level.

The effect of pregnancy on myelodysplastic syndrome is controversial. Siddiqui^7^ et al have reported cases of conversion of myelodysplastic syndrome to AML during pregnancy, while Ikeda,^8^ Gidiri et al^9^ believe that pregnancy itself does not affect the outcome of myelodysplastic syndrome, and the hematological outlook of the patient can also be improved after delivery. In our case, the patient had been self‐managing her condition using nonprescribed drugs for a long time, and her conditions had been quite stable during this time. However, she repeatedly experienced nosebleed and gingival bleeding during her pregnancy, which indicate that her condition may have been exacerbated by pregnancy. After being discharged, the patient did not receive further treatment, and her condition was relatively stable in the telephone follow‐up 42 days later. Therefore, we speculate that this case may be illustrating pregnancy‐induced exacerbation of MDS.

The American Society of Hematology recommends that platelets of patients with idiopathic thrombocytopenia be maintained at a minimum of 50 × 10^9^/L before and during delivery.^10^ Steensma et al^11^ suggest that patients with myelodysplastic syndrome should maintain more than 50 × 10^9^/L platelets if undergoing a cesarean section and 20‐30 × 10^9^/L platelets for a vaginal delivery. In the present case, our patient's platelet count fluctuated between 1‐2 × 10^9^/L, multiple hemorrhages occurred all over the body and became increasingly severe, and repeat platelet transfusions were ineffective. With a vaginal delivery, the induction of labor may last for a long time and both the use of drugs for labor induction and the abdominal pressure experienced during the labor process may cause visceral and intracranial hemorrhage. Furthermore, the hemostasis of bleeding in the birth canal would also have been difficult to treat. On the contrary, hemostatic difficulty, massive bleeding, pelvic hematoma, and subcutaneous hematoma are all likely to manifest during a cesarean operation. The patient had severe anemia and an extremely low tolerance for blood loss. Our goal was to increase her platelet count to 20 × 10^9^/L and her hemoglobin concentration to 4 g/dL to begin induction of labor and attempt vaginal delivery, after consideration of multiple factors such as the risk of bleeding, cervical maturity, pelvic condition, mental state of the pregnant woman, fetal size, and others. However, in this particular case, the patient may have had anti‐blood‐cell and anti‐platelet antibodies, which posed difficulties in stabilizing the patient through conventional transfusion protocols alone. There have been reports in the literature stating that cross‐matched platelets can be used for immune refractory thrombocytopenia,^12^ and the American Society of Hematology recommends prednisolone as the first‐line drug for pregnancy complicated by myelodysplastic syndrome.^10^ Hence, we prescribed the patient to take 30 mg of oral prednisolone every day and transfused cross‐matched platelets simultaneously to rapidly increase her platelets for induction of labor. At the same time, to prevent bleeding, anything that causes abdominal pressure should be avoided and the patient should be given effective labor analgesia during delivery (epidural analgesia can be administered if platelet count is above 50 × 10^9^/L^13^).

The onset of myelodysplastic syndrome is insidious, its clinical manifestations are atypical, and treatment is often intractable. Therefore, early diagnosis is especially important. In the presence of unexplained anemia, fever, and a tendency to bleed during pregnancy, hematologic diseases should be considered as a possible etiology. When a patient with this condition presents to the clinic, it is imperative that the medical staff first perform a routine blood test on the patient, including the leukocyte differential count. It may also be necessary to perform a bone marrow puncture to confirm the diagnosis. They should carefully determine whether there is any indication to continue the pregnancy. Altogether, this case highlights some of the challenges faced in the management of patients with severe and refractory immune thrombocytopenia during pregnancy.

## CONFLICT OF INTEREST

The authors declare that they have no competing interests.

## AUTHOR CONTRIBUTIONS

CP and TC: were the most responsible doctors of this patient. They designed the study and drafted the manuscript. QY: was the principal supervisor reviewed and edited the manuscript. All three authors read and approved the final manuscript.

## ETHICAL APPROVAL

This research does not involve human subject trial. Written informed consent for publication of this case study has been obtained from the legally authorized representative of this patient.

## CONSENT TO PUBLISH

Informed consent was obtained from all participants.

## Data Availability

All data generated or analyzed during this study are included in this published article, and original data can be requested from Changsheng Peng.
